# Photoresponsive Biomimetic Functions by Light-Driven Molecular Motors in Three Dimensionally Printed Liquid Crystal Elastomers

**DOI:** 10.1021/jacs.4c01642

**Published:** 2024-05-10

**Authors:** Guiying Long, Yanping Deng, Wei Zhao, Guofu Zhou, Dirk J. Broer, Ben L. Feringa, Jiawen Chen

**Affiliations:** † SCNU-UG International Joint Laboratory of Molecular Science and Displays, National Center for International Research on Green Optoelectronics, South China Normal University, Guangzhou 510006, China; ‡ Stratingh Institute for Chemistry, University of Groningen, Nijenborgh 4, Groningen 9747 AG, The Netherlands; § SCNU-TUE Joint lab of Device Integrated Responsive Materials (DIRM), Guangdong Provincial Key Laboratory of Optical Information Materials and Technology & Institute of Electronic Paper Displays, South China Academy of Advanced Optoelectronics, South China Normal University, Guangzhou 510006, China; ∥ Stimuli-responsive Functional Materials and Devices, Department of Chemical Engineering and Chemistry, Eindhoven University of Technology, Eindhoven 5600 MB, The Netherlands

## Abstract

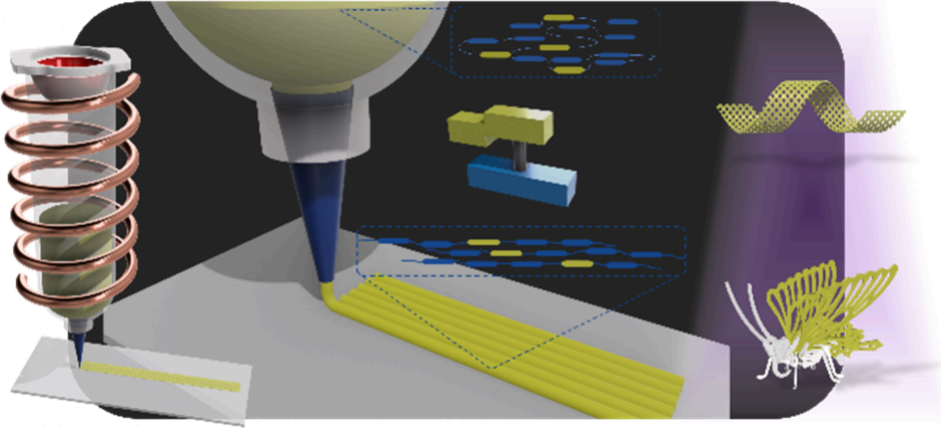

Despite the fascinating developments in design and synthesis of artificial molecular machines operating at the nanoscales, translating molecular motion along multiple length scales and inducing mechanical motion of a three-dimensional macroscopic entity remains an important challenge. The key to addressing this amplification of motion relies on the effective organization of molecular machines in a well-defined environment. By taking advantage of long-range orientational order and hierarchical structures of liquid crystals and unidirectional rotation of light-driven molecular motors, we report here photoresponsive biomimetic functions of liquid crystal elastomers (LCEs) by the repetitive unidirectional rotation of molecular motors using 3D printing. Molecular motors were built in the main chain of liquid crystals oligomers to serve as photoactuators. The oligomers were then used as the ink, and liquid crystal elastomers with different morphologies were printed. The obtained LCEs are able to conduct multiple types of motions including bending, helical coiling, closing of petals, and flipping of wings of a butterfly upon UV illumination, which paves the way for future design of responsive materials with enhanced complex actuating functions.

## Introduction

Motion is of great importance in nature as it sustains a broad range of essential functions for all living systems.^1,2^ These motions are operated by biological molecular machines that convert chemical energy upon external stimuli and amplification along multiple length scales eventually allow execution of specific activities and functions, which are accomplished with high efficiency, selectivity, and complexity under precise control. Representative examples are the unidirectional rotation of ATP synthase to generate the required energy, the closure of Venus Flytrap to capture insects, and flipping of wings sustain flying animals. Inspired by these sophisticated natural systems, scientists have been designing and constructing synthetic molecular machines that utilize chemical, photochemical, electrical, and thermal energy input to achieve movement or distinct mechanical operations.^3−13^ These molecular machines have been integrated in well-organized structures for the realization of a large variety of specific functions. Illustrative examples include molecular switches,^14−17^ rotors,^18−21^ chemical multitasking catalysts,^22,23^ nano cars,^24^ synthesizers,^25,26^ molecular shuttles,^27−29^ self-sorting machines,^30,31^ transporters,^32,33^ and pumps.^34−36^ Translating molecular motion along multiple length scales in order to induce motion at macroscopic dimensions, however, remains a key scientific issue. One of the most practical approaches are to incorporate molecular machines inside a material through supramolecular self-assembly or covalent bonding. Taking advantage of embedding intrinsic artificial molecular machine functions into materials, a large range of multicomponent responsive materials have been developed,^37−39^ enabling dynamic, mechanical, and smart materials.

Among all of the stimuli-triggered responsive materials, the use of light as the external stimulus and as a clean energy source has desirable attributes. As light can be precisely controlled with short response time and produces no waste and high spatial and temporal precision can be reached, photoresponsive smart materials have seen important developments.^40−43^ Most systems are based on two typical molecular photoswitches: azobenzene^44,45^ and diarylethene.^46,47^ Azobenzene undergoes trans–cis isomerization by irradiation with UV light and returns to its original stable position by either illumination at a different wavelength or by heat. Similarly, diarylethenes can undergo ring opening or closure reactions after being irradiated at the appropriate wavelength of light. The large differences of molecular configurations after irradiation lead to changes in the shape, polarity, and electrical properties of the entire molecule, which serves as the key to photoresponsive dynamic functions of smart materials based on these two types of molecular photoswitches. Despite these advances, the development of systems in which molecular movement is translated and amplified along multiple length scales to induce macroscopic motion as a basis for actuator materials has been limited. Facing the challenge to achieve multiple distinctive autonomous motions in a soft material powered by light, we envisioned that our light-driven rotary motors based on overcrowded alkenes offer unique opportunities to achieve complex mechanical movements. The inherently chiral molecular motors can rotate unidirectionally triggered by light in a noninvasive manner.^3,10,18,48−50^ The rotary cycle of motors involves not only geometrical changes of the molecule but also helicity changes, which distinguishes motors from most other molecular switches. Molecular motors have been applied to achieve macroscopic functions, including dynamical tuning of wettability of surfaces^51^ and photoactuation of a supramolecular muscle.^52^ In addition, liquid crystals (LC) were employed as molecular motors can organize in line within LC molecules and the long-range orientational order of LC promotes amplification of molecular motion of the doped molecular motor from the nanoscale upward. Our previous studies have shown that molecular motor is compatible with liquid crystals, both in noncovalent^53−55^ and covalent systems,^56,57^ while retaining its rotary motion. As a proof of concept, motors were embedded in a liquid crystal network (LCN), serving as cross-linkers, photoactuators, and chiral dopants at the same time. The corresponding two-dimensional polymeric film was then prepared, and the disorder of the LC materials created by rotary motion of the motor leads to anisotropic deformation of the LC film, which results in bending and helical twisting.^56,57^ Despite the encouraging results, responsive LCN is limited by its preparative approach. Two glass plates are usually glued together with a certain space to form a glass cell and the LC mixture of monomers are then filled inside this cell. The predefined patterns on both surfaces of the glasses determine the orientation of LC monomers, usually resulting in parallel, splay, or helical alignment. The mixtures are subsequently polymerized and the cell is opened afterward, generating a solid LCN film. This approach is limited by the size of the glass cell and the space between both glasses as larger spacing inevitably reduces the degree of orientation of the LC monomers, which leads to much less deformation of the LCN. Therefore, an LCN thin film (<100 μm) is usually prepared in order to realize profound responsive functions, which greatly limits its further application as photoactive systems where larger sizes or thicknesses are not guaranteed.

**Figure 1:**
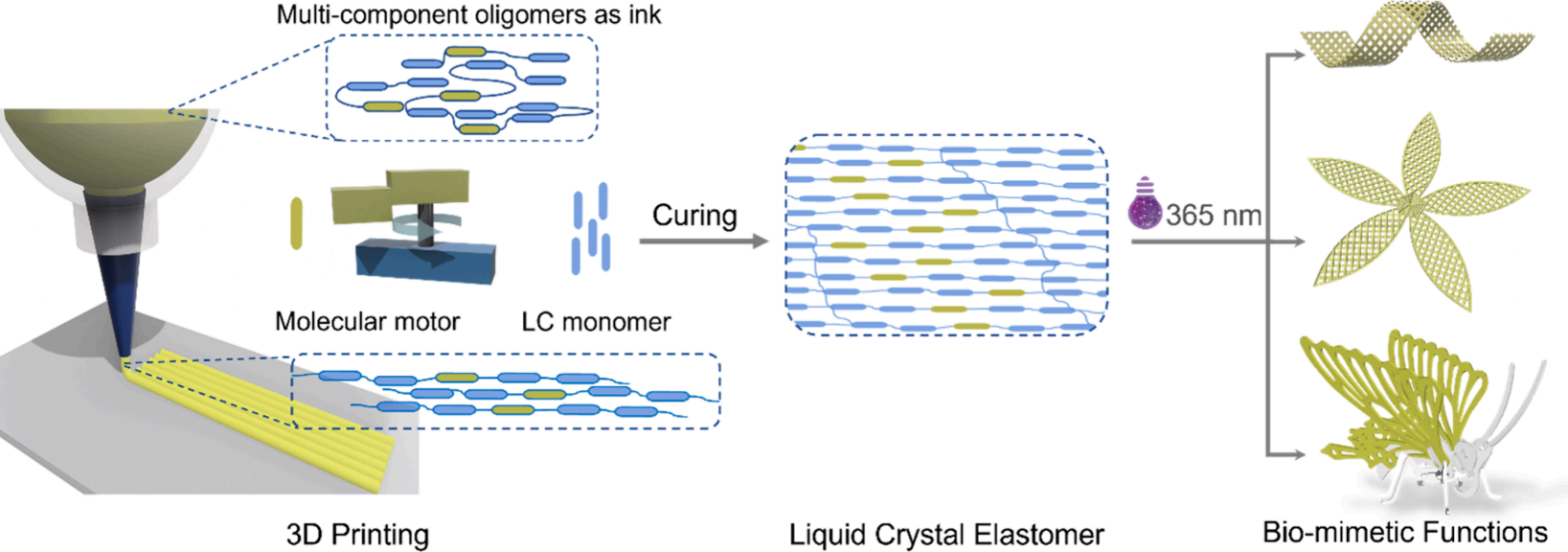
Representative scheme for the programmable construction of LCE containing molecular motors by the 3D printing technique. Liquid crystal mixtures doped with light-driven rotating motors are prepared as oligomers to serve as inks for 3D printing. The printed LCE samples are able to perform bending, controlled helical motion, and biomimetic functions upon UV light irradiations.

Alternatively, the use of liquid crystal elastomers (LCEs) represents another important approach for the construction of adaptive soft materials. The preparation of LCEs first involves chemical connection of liquid crystal monomers to form oligomers, which is followed by network cross-linking, resulting in a looser network structure that exhibit viscoelastic and non-Newtonian fluid properties.^58−66^ Unlike LCN that typically has a glass transition temperature (i_g_) above r.t. and a high cross-link density, LCE has a larger strain-at-break value and low elastic modulus at r.t., thus providing more space for ordered-disordered transitions,^67−69^ enabling large and reversible deformations when triggered by certain stimuli. More importantly, besides the cell preparation approach, LCE can also be rapidly fabricated by additive manufacturing method, such as 3D printing to create complex objects with substantial sizes.^64,70−78^ When an LCE is prepared by 3D printing, a viscous ink composed of non-cross-linked liquid crystal oligomers is extruded through a printing nozzle and the liquid crystal oligomers are spontaneously aligned along the printing path by the shear stress generated during the extrusion process. By design, the local alignment of the liquid crystals can be well organized and a system with multifunctional behavior is created. To date, thermally active LCE systems fabricated by 3D printing have shown large, reversible shape changes,^79−87^ while photoresponsive LCEs are still largely unexplored.^88−90^ By taking advantage of the long-range orientational order and hierarchical structures of LCE, we envisioned that the repetitive unidirectional rotation of molecular motors can be transformed and amplified along all length scales, resulting in photoactive three-dimensional objects with advanced structural complexity and sophisticated mechanical motions (Figure 1). In this study, light-driven molecular motors were first incorporated in the main chain of the LC oligomers by thiol–Michael addition. The resulting oligomers exhibit suitable thermal and rheological properties and, therefore, are employed as the ink for 3D printing. By predefined printing paths, photoactive LCE objects with a large variety of morphologies and various sizes, incorporating racemic or homochiral motors, were prepared.

Using these systems, biomimetic functions on demand, including helical coiling, closure of petals of flowers, and flipping of wings of a butterfly, were realized.

## Results and Discussion

### Photoresponsive Ink for 3D Printing

**Figure 2:**
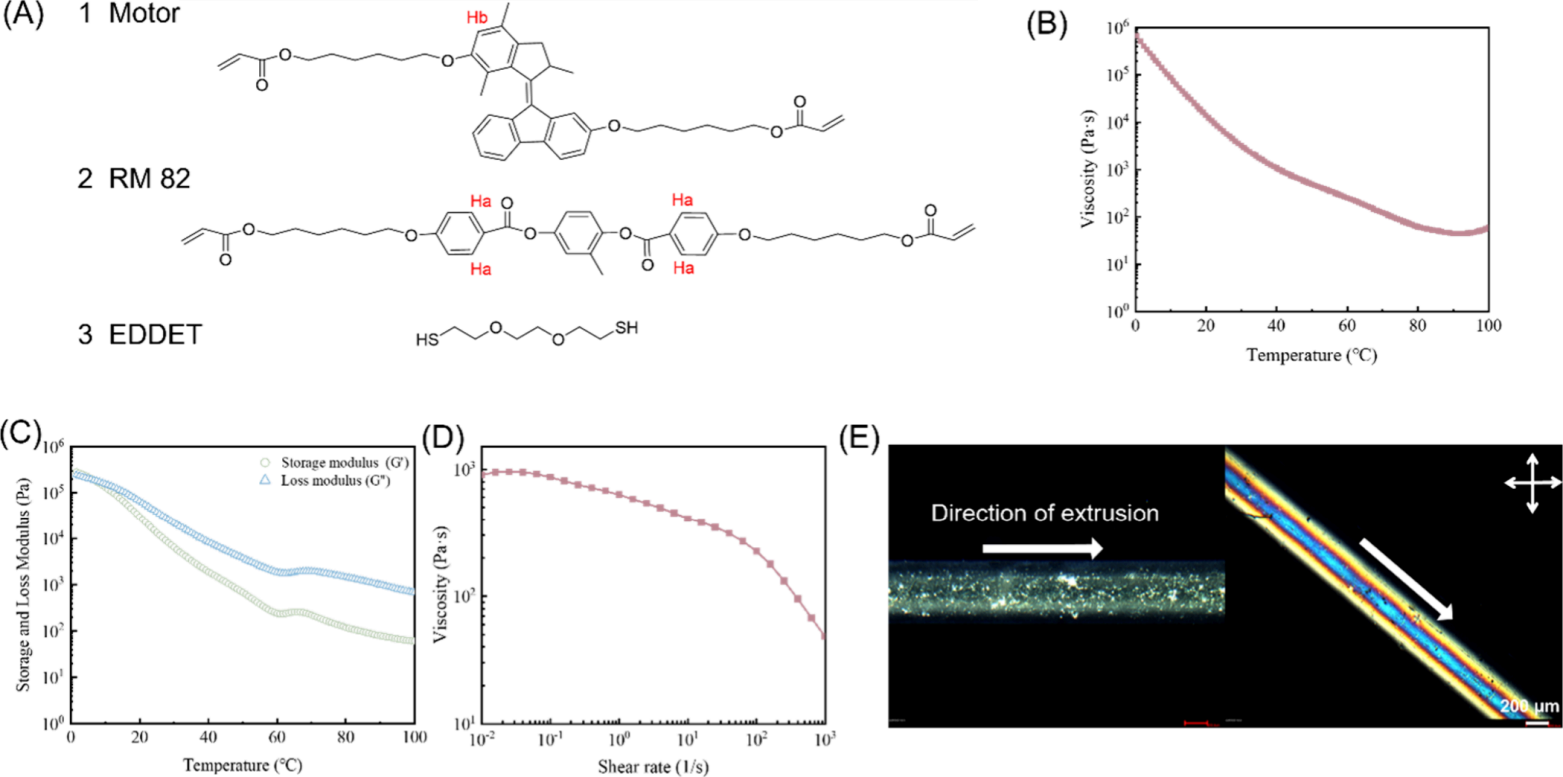
(A) Chemical structures of motor, liquid crystal monomer RM 82 and chain extender EDDET for the synthesis of liquid crystal oligomers. (B–D) Rheological characterization of the obtained LC oligomers. (B) Liquid crystal oligomers exhibit a change in viscosity that decreases with increasing temperature. (C) Changes of the storage modulus (*G*′) and loss modulus (*G*″) of inks at different temperatures. (D) Variation of the viscosity of the ink with different shear rates at a fixed temperature of 45 °C. (E) POM images of the 3D printed oligomers. The white bidirectional arrow represents the direction of the polarizer and the analyzer. The white unidirectional arrow indicates the direction of printing.

A light-driven molecular motor **1** (Figure 2A) was selected as the key component of photoresponsive ink for 3D printing. First, it consists of a cyclopentene upper and a fluorene lower half, which are connected with a carbon–carbon double bond. The central overcrowded olefinic bond serves as the rotary axle, and rotary motion of **1** has been studied in details in our previous report.^57^ Motor **1** is able to undergo a full 360° unidirectional rotary motion upon irradiation with a rotary speed of 1 min at rt, which fits our purpose for preparation of a fast responsive system. In addition, two acrylate moieties are placed at both sides of the motor to enable the copolymerization in the liquid crystal elastomers with a C-6 carbon spacer installed between the motor core and the acrylate groups to provide enough free space for the motor to rotate inside the LC polymer. Next, motor **1**, liquid crystal monomer RM 82 and chain extender 3,6-dioxa-1,8-octanedithiol (EDDET), whose chemical structures are shown in Figure 2A, were mixed to prepare main-chain LC oligomers as the ink. To induce the thiol–acrylate Michael addition in the presence of base, the mixture was dissolved in dichloromethane, triethylamine (TEA) was added dropwise to the solution, and the reaction was carried out overnight at 40 °C. However, the above condition only worked at small scale, i.e., less than 1 g. When the reaction was carried out on a large scale, i.e., more than 1 g, the degree of polymerization of the resulting oligomers and their properties such as viscosity were not consistent. Therefore, 2,3,4,6,7,8,9,10-octahydropyrimido[1,2-*a*]azepine (DBU) was used instead of TEA and to our delight, the conversion was enhanced to become completed within 2 h. Differential scanning calorimetry (DSC) and ^1^H NMR studies of the obtained liquid crystal oligomers prepared by both approaches are shown and compared in Figure S4 and no significant differences were found, indicating DBU was superior to TEA as it ensures the preparation of the main-chain oligomers at large scales with lower reaction time.

To further investigate the effect of chain extender on the properties of the prepared oligomers, different molar ratios of acrylate and chain extender were screened (1:0.60, 1:0.75, 1:0.80, 1:0.85, 1:0.90), and ^1^H NMR and DSC studies were employed to evaluate the effect (Figures S2 and S3). The results show that the phase transition temperature of the oligomer decreases, and the viscosity of the system increases as the amount of thiol increases. To ensure alignment of the liquid crystal and the quality of the printed pattern, the 3D printing working temperature is usually 10 °C lower than the phase transition temperature of the ink.^69^ Therefore, in our present study, a molar ratio of 1:0.90 (acrylate: thiol) was employed. DSC and polarized optical microscopy (POM) show that the temperature at which the material changes from the nematic to isotropic phase (*T*_N/I_) is 68 °C (Figure S4C). The resulting oligomer was also studied by ^1^H NMR (Figure S1). The signals at 8.17 ppm were assigned to be protons Ha of the liquid crystal monomer RM 82, and the singlet at 7.44 ppm was the characteristic peak of proton Hb of the lower half of motor **1**,^55^ while the peaks between 6.50 and 5.80 ppm can be assigned as the signals of acrylate moieties. Based on the integration of corresponding units, the degree of polymerization of the obtained oligomer was calculated to be 9.98. Furthermore, gel permeation chromatography (GPC) (Figure S5) showed that the number-average molecular weight (*M*_n_) of these oligomers was calculated to be ≈4000 g/mol, the weight-average molecular weight was ≈18000 g/mol, and the polydispersity index (PDI) was determined to be 4.61.

With the LC oligomer in hand, we further tested the rheology as viscosity is a crucial property for the printing inks. As shown in Figure 2B, the motor-based LC oligomer exhibited a temperature-dependent viscosity change. The viscosity dropped sharply when the temperature increased in the range 0–45 °C. In the temperature range of 45–80 °C, the viscosity decreased at a slow rate with the increase of temperature, and the viscosity curve leveled off or increased slightly when the temperature was above 80 °C. As shown in Figure 2C, with a fixed stress of 1% and a constant frequency of 1 Hz, the storage modulus (*G*′) and loss modulus (*G*″) curves of the LC oligomer showed rapid decreases when the temperature was raised. The loss modulus was always lower than the storage modulus after 10 °C, but the decrease of the loss modulus is not as much as that of energy storage modulus, indicating that the ink tends to be a viscoelastic liquid.^91,92^ Then the LC oligomer was placed for the rotational test at a fixed temperature of 45 °C. As shown in Figure 2D, the viscosity of the ink decreased when the shear rate was increased, exhibiting significant shear thinning property. This property is crucial as it ensures the ink to be successfully extruded through the printing nozzle without clogging. In addition, chain reorientation can take place at high shear rates, aligning LC molecules internally within a certain order.^82,88,93^ The observed typical shear-thinning and temperature-responsive rheological properties of the prepared LC oligomer proved highly suitable as the ink for 3D printing (Figure 2, Figure S6).^61,66^

For the printings, in order to optimize the LC materials and thereby the quality of the printed pattern, we tested the correlation between the molecular orientation of LC materials and the printing speed. At a fixed temperature (45 °C), extrusion pressure (3 bar), nozzle diameter (410 μm), and distance between the nozzle and the printing substrate (0.6 mm), different printing speeds (4–13 mm/s) were screened. Parallel oriented LCE strips were printed and investigated under POM. The strips appeared bright when the crossed polarizers are oriented at 45°/135° and dim when they are oriented at 0°/90° (Figure S9), indicating that the orientation of the printed LCE is along the printing direction. As the printing speed increased, strips with smaller diameters were obtained, which indicated a higher degree of alignment within the LCE according to the POM image. However, when the speed kept increasing, the viscosity of the LC mixture mismatched the extrusion speed, generating inhomogeneous LCE materials. After careful screening, the optimal printing speed was set at 10 mm/s. The POM image of the LCE strip printed at this speed is shown in Figure 2E, indicating good orientation of the LC materials. Furthermore, it is confirmed by WAXS that the internal alignment of the LCE objects obtained by the 3D printing technique shows an ordered state (Figure S10).

### Photoresponsive Motion of 3D-Printed LCE Films

To investigate the photodynamics of the LCE films, we first designed an LCE film (5 mm × 30 mm) with parallel orientation and printed onto a glass substrate coated with poly(vinyl alcohol) (PVA) as a sacrificial layer. The newly printed film was exposed to blue light (455 nm) for 2 h to ensure the completed cross-linking of the acrylate moieties.

**Figure 3:**
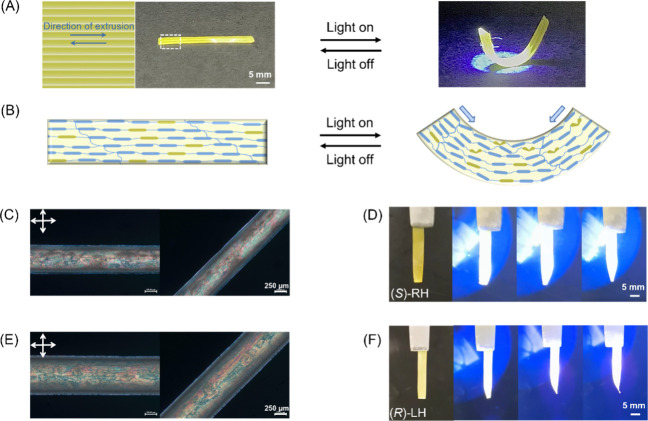
Phototriggered actuations of the 3D-printed LCE film containing racemic and enantiomerically pure motor **1**. (A) Bending motion of the LCE film under 365 nm light. The blue arrows show the direction of extrusion during printing. (B) Schematic diagram of the deformation. The thick arrows represent the direction of shrinkage. (C) POM images of LCE strip with 1 mol % of (*S*)-motor. (D) LCE strip with (*S*)-motor showed right-handed helical motion upon UV irradiation. (E) POM images of LCE strip with 1 mol % of (*R*)-motor. (F) LCE strip with (*R*)-motor showed left-handed helical motion upon UV irradiation. The light intensity is 100 mW/cm^2^.

DSC and IR studies confirmed the complete polymerization of the resulting film (Figures S7 and S8). The PVA layer was then dissolved in water and the film could be isolated from the glass substrate and dried under air. The film was subsequently submitted to UV light (365 nm) irradiation with an intensity of 100 mW/cm^2^ and showed actuation toward the light source. The saturated bending motion was completed within 2 s, and the film regained its initial position instantly after the light was switched off (Figure 3A, Movie S1). In addition, several cycles could be performed by subsequent on and off switching of the light and the system did not show significant fatigue. When the film was stimulated by light, the rotary motion of motors inside the LCE took place, causing the anisotropic contraction of the film and, as a consequence, induced bending of the film toward the light source (Figure 3B). To further confirm that the observed actuation of the LCE film containing motor **1** is predominantly due to the rotation and change in shape of the motor, the LCE film was brought to water to suppress heating of the sample. To our delight, actuation of the sample after UV irradiation was observed with a similar speed as that in air (Figure S11, Movies S2 and S3), which unambiguously shows deformation of the film was driven by rotary motion of the embedded motor rather than a photothermal effect.

Next, an enantiomerically pure motor was applied to the system. Due to the unique axial chirality of motor **1**, it can act as a chiral dopant, inducing the LC system to produce a cholesteric phase. Motor **1** is a strong chiral dopant with an HTP value of ±115 μm^–1^.^56^ Therefore, in the present study, we prepared the cholesteric LC ink by preliminary cross-linking the enantiomeric motor (containing 1 mol % of (*R*)-1 or (*S*)-1) with liquid crystal monomer and chain extender (for details of preparation, see the SI). In order to maintain the ink in the cholesteric phase, the temperature inside the printing syringe needs to be set higher than the *T*_N/I_.^69,89,90^ Based on the result of DSC measurement (figure S4A), a printing temperature of 90 °C was employed to reduce the viscosity of the ink. In addition, the temperature of the holding substrate was set to 40 °C, which is below T_N/I_, and the final printing speed was set to 6 mm/s, somewhat lower than that for the achiral ink, to allow the LC materials to form a cholesteric phase smoothly. Under the optimized condition, LC strips with embedded (*R*)-1 and (*S*)-1 were prepared, respectively, whose POM images are shown in Figure 3C,E. As no significant changes were observed when the samples were parallel or at 45° to the polarizer or analyzer under POM, this suggests that the cholesteric phase had been successfully formed. The obtained LC stripes were then exposed to UV light (365 nm) with an intensity of 100 mW/cm^2^. The stripe with (*R*)-1 showed left-handed helical motion while the stripe with (*S*)-1 displayed right-handed helical motion (Figure 3D,F, Movies S4 and S5). Both stripes reached their saturated states within 2 s and recovered to their original states after the UV-lights. The above experimental data indicate the unique features of the light-driven molecular motor as all key functions are embedded in one single molecular structure including photoactuator, directional motion, multiple helical states, and intrinsically chiral dopant. The phototriggered unidirectional rotation of motor at nanoscale is amplified along multiple length scales and ultimately results in the controlled right-or-left handed helical coiling of the 3D printed LC stripe.

### Photoresponsive Biomimetic Functions of 3D-Printed LCE

**Figure 4:**
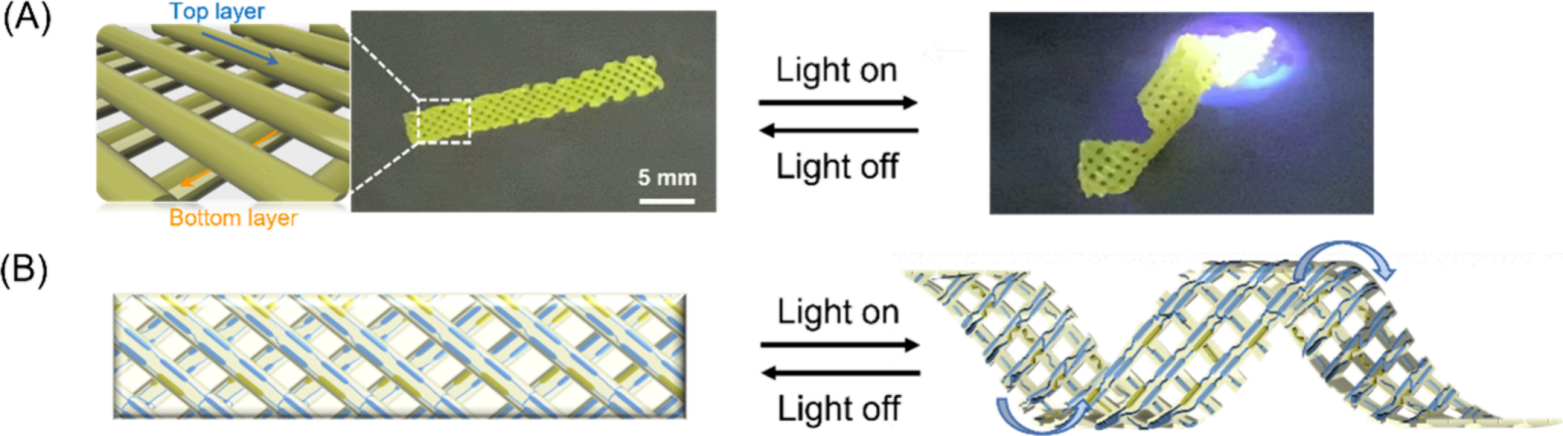
(A) Helical deformation of a bilayer LCE stripe under 365 nm light; the arrows show the direction of extrusion during printing, with the top and bottom layers printed at ±45° with respect to the long axle of the stripe. (B) Schematic diagram of the out-of-plane deformation of a bilayer LCE stripe due to uneven spatial curvature. The light intensity is 100 mW/cm^2^.

In order to achieve more complex motion, we take advantage of the 3D printing technique since the printing parameters, including printing directions, layers, and patterns, can be preprogrammed by design. A bilayer approach was explored as it is envisioned that each layer containing motors has been shown to actuate upon light irradiation, and the combination of two layers with a certain pattern can lead to more advanced deformation of the LC materials. In the present study, a bilayer strip with 45°/135° to the long axle was designed, as shown in Figure 4A (Movie S6). An internal spacing of each layer was set to be 1 mm to ensure enough space for deformation, and a total size of the strip was 5 mm × 30 mm. The same condition was applied as the one used for printing of the LCE with racemic motors (for details of preparation, see the SI). The first layer was printed at an angle of 45° to the long axle of the stripe, and the molecular orientation of the printed LC materials was aligned along with the printing direction by shear stress. After printing, the sample was exposed to blue light (455 nm) at a power of 200 mW/cm^2^ for 300 s to further fix the internal orientation of the obtained LC stripe. A second layer was subsequently printed on top of the first layer with an angle of 135° to the long axle of the stripe, employing the same printing parameters. The obtained bilayer sample was again placed under blue light (455 nm) for 2 h and was turned over every 30 min to ensure full cross-linking. Subsequently, the film was separated from the glass substrate by dissolving the PVA layer. As anticipated, the bilayer stripe displayed a helical motion under UV light (365 nm) irradiation as shown in Figure 4A. The helical coiling of the stripe was attributed to the bilayer design. The LCE strip containing motors with a uniaxial orientation is able to perform contraction and bending deformations along a predefined direction which is determined by orientation of the LC materials as shown in Figure 3A. When two identical layers were combined with a certain angle, each layer actuated in its preferred direction, leading to deformation of the LC stripe in different directions. Incompatible strains occur due to the uneven spatial distribution of bending curvature, which ultimately resulted in out-of-plane deformation of the stripe, as shown in Figure 4B. This approach set the stage for further construction of biomimetic responsive objects.

**Figure 5:**
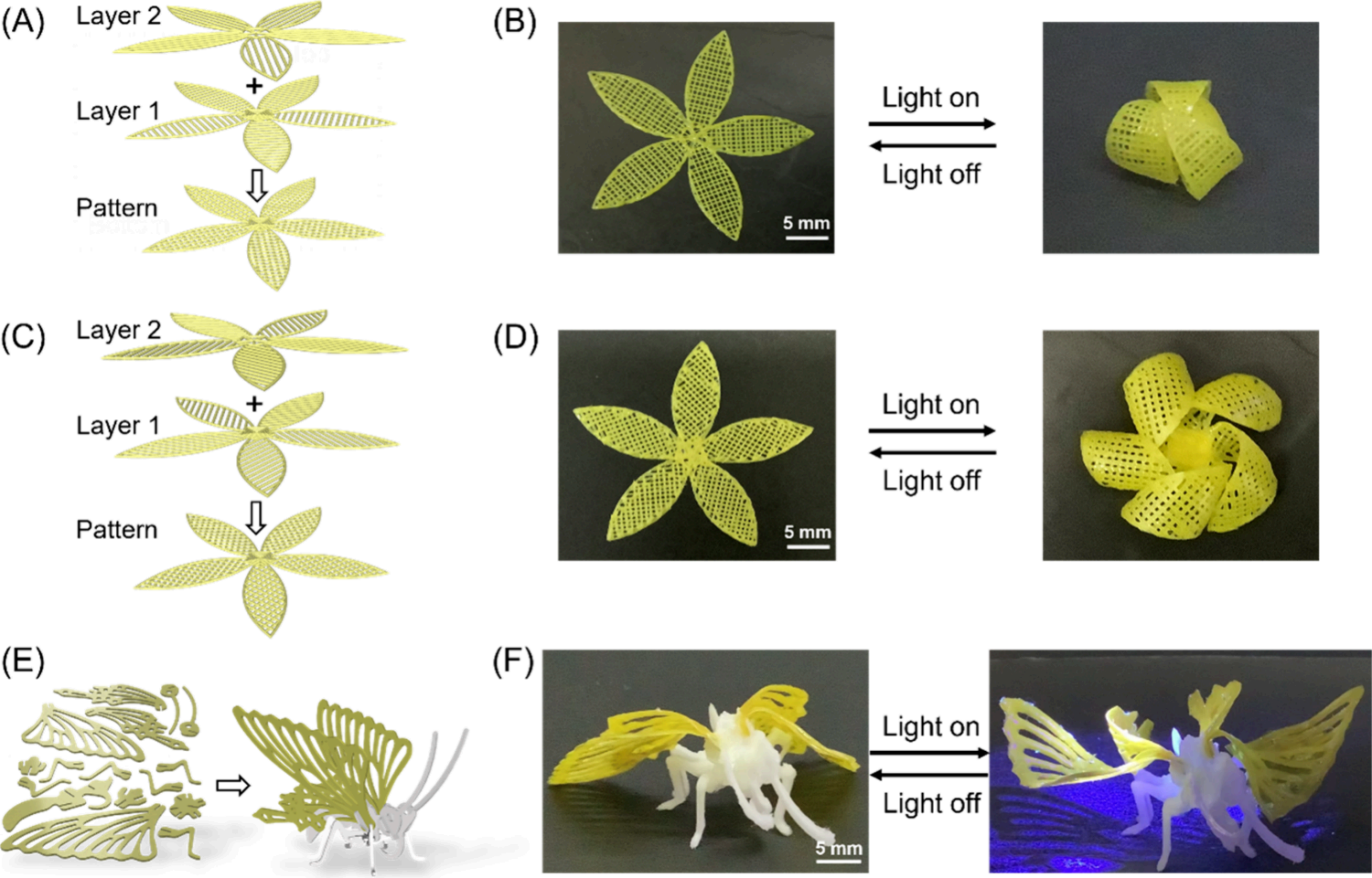
Photoresponsive biomimetic functions of 3D printed LCE. (A) Schematic diagram of bilayer LCE flowers. The angle between the printing paths of the two layers is 0°/90° to the long axle. (B) When the flower is stimulated by UV light, all of the petals display bending deformation, representing closure of a flower. (C) Schematic diagram of a bilayer flower with an angle of 45°/135° between the two printing layers. (D) 3D-printed flower shows helical coiling when placed under UV light. (E) Schematic diagram of a 3D butterfly. (F) Deformation of the printed butterfly under UV light mimics flipping of wings. The white part is built from PCL materials, and the yellow part comprises the photoresponsive LCE material. The light intensity is 130 mW/cm^2^.

Two photoresponsive flowers were then designed and printed (Figure 5). In the first case, the petal size was set to be 5 × 15 mm and consisted of a double layer with a printing direction of 0°/90° to the long axle, respectively. An internal line spacing of 1 mm was installed between two layers, as shown in Figure 5A. The predefined flower was printed, cured, and dried (for details of preparation, see the SI). The resulting sample was subsequently submitted to UV irradiation, and the petals displayed full closure within 60 s and returned to its initial opening state when the light source was switched off (Figure 5B, Movie S7), mimicking the open and closed of natural flowers. Interestingly, when the printing directions of the petal were altered to 45°/135° with respect to the long axle for the double-layer structure (Figure 5C), UV light irradiation triggered coiling, resulting in helical closures of the petals (Figure 5D, Movie S8). It should be emphasized that the direction and shape of petal closing are highly dependent on the preprogrammed printing pathway, i.e., the number of layers and the angle between the layers.

Finally, we tested the possibility of constructing a real photoresponsive 3D object of substantial size. A hybrid system that mimics a butterfly was designed, as shown in Figure 5E. Polycaprolactone (PCL) was employed to build the main body as it has high modulus and is able to support the 3D model, while the photoactive LCE containing molecular motors serve as the flapping wings. PCL with a molecular weight of 50,000–90 000 Da was first dissolved in dichloromethane at 38 °C and can be directly used as the inks. The printing speed was adjusted to 15 mm/s with an extrusion pressure of 2 bar. The temperature of the glass substrate was tuned to be 40 °C as it can enable evaporation of the solvent (DCM) in line with the extrusion process, leading to quick solidification of the sample (Table S13). Next, the wings were prepared by the LCE containing motor **1** with a parallel orientation and connected to the main body to make the 3D object ready with a size of 55 × 55 × 30 mm. In accordance with our preset deformation, the wings were able to reversibly flip under on–off illumination of UV light (Figure 5F, Movie S9), mimicking the wing movement associated with the flying motion of a butterfly.

## Conclusions

Responsive soft polymeric materials that allow multiple distinct and well-defined motions powered by light are of major importance to enable the development of complex mechanical actuating systems. The key to addressing this challenge relies on the incorporation and effective organization of photoactive molecular machines in a well-defined environment, which enables cooperative amplification and directional motions along all length scales to induce anisotropic deformation of macroscopic objects. Here, we show photoresponsive biomimetic functions by programmable embedding of light-driven molecular motors in LCE by the 3D printing approach. Motor **1** was installed in the main chain of LC oligomers and after screening of the base used and ratio of thiol and arylate moieties, oligomers with printable thermal and rheological properties could be obtained. By taking the optimal printing condition, LCEs with a large variety of morphologies were prepared. The molecular orientation of the LC materials was aligned along with the printing direction by shear stress and therefore ensured the hierarchical organization of motors in the LCE actuator. Phototriggered actuation of the obtained LCE was observed, and control experiments were conducted to confirm that the deformation can be predominantly attributed to the rotary motion of motors. Besides fast bending motion, LCEs with enantiomeric pure motors displayed controlled helical coiling, the direction of which depends on the handedness of the motor. In addition, a bilayered approach was applied to construct LCEs with advanced functions. Phototriggered closure of petals of a flower was achieved, and the direction and shape of petal closing are highly dependent on the predefined printing pathway, i.e., the number of layers and the angle between the layers. Furthermore, a hybrid polymer system was employed to construct a three-dimensional butterfly. PCL was used to support the main body and photoactive LCE was used as the wings. Flapping motion of the wings could be realized under UV illumination. This study shows how rotary motion of molecular motors can be programmed and amplified to achieve biomimetic functions in three-dimensional objects of substantial sizes, which paves the way for the future design of advanced responsive and adaptive soft materials with more complicated mechanical motions.

## Supporting Information Available

Preparation processes, characterization data (including ^1^H NMR, DSC studies, GPC studies) for all materials and explorations related to 3D printing parameters (PDF)
Bending motion and curling motion of LCE strips and bionic motion of LCE actuators for petal opening and closing and butterfly flapping wings (MP4, MP4, MP4, MP4, MP4, MP4, MP4, MP4, MP4)
