# Supplementary material for: Photoresponsive Biomimetic Functions by Light-Driven Molecular Motors in Three Dimensionally Printed Liquid Crystal Elastomers
Source: J Am Chem Soc. 2024 May 10;146(20):13894–902. doi: 10.1021/jacs.4c01642 (PMC11117400; doi:10.1021/jacs.4c01642)
Supplement: Supplementary file 1 — ja4c01642_si_001.pdf [file ja4c01642_si_001.pdf]

Supplementary Information for

Photo-responsive bio-mimetic functions by light-driven molecular motors in 3D printed liquid crystal elastomers

Guiying Long[1,2], Yanping Deng[1], Wei Zhao[3], Guofu Zhou[1,3], Dirk J. Broer[3,4], Ben L. Feringa*[,1,2], Jiawen Chen*[,1]

1 SCNU-UG International Joint Laboratory of Molecular Science and Displays, National Center for International Research on Green Optoelectronics, South China Normal University, Guangzhou 510006, China.

2 Stratingh Institute for Chemistry, University of Groningen, Nijenborgh 4, 9747AG Groningen, The Netherlands.

3 SCNU-TUE Joint lab of Device Integrated Responsive Materials (DIRM), Guangdong Provincial Key Laboratory of Optical Information Materials and Technology & Institute of Electronic Paper Displays, South China Academy of Advanced Optoelectronics, South China Normal University, Guangzhou 510006, China.

4 Stimuli - responsive Functional Materials and Devices, Department of Chemical Engineering and Chemistry, Eindhoven University of Technology, Den Dolech 2, Eindhoven, 5600 MB, The Netherlands.

Correspondence to Jiawen Chen and Ben L. Feringa

Experimental Section

General remarks

Characterization: ^{1}H-NMR spectra were recorded by Bruker Advance III HD (600 MHz). The corresponding chemical shifts were reported in δ values (ppm) relative to deuterochloroform (CDCl3; ^{1}H δ=7.25). DSC (Differential Scanning Calorimetry) tests were used to determine the oligomer phase transition temperature and the degree of polymerization of the LCE using a TA Instruments DSC Q1000. Irradiation experiments were performed using an LED lamp (Thorlabs) at 365 nm. All optical phenomena of LCE and CLCE were observed and recorded by polarized optical microscopy (POM; DM2700p, Leica). Enantiopure motors were obtained by chiral HPLC (ODH; isopropanol: heptane = 1:99, flow rate 1 mL/min). The spectrometer used in the FTIR spectroscopy tests was a Nicolet 670, with a spectral resolution of 0.09 cm $^{-1}$ and providing 105 times/s fast scans and time-resolved spectra of better than 10 nanoseconds. Wide-Angle X-Ray Scattering (WAXS) performed the measurements using a Xeuss 3.0 model configured with an 8.05 KeV X-ray copper target producing a wavelength of 1.54189 Å and an Eiger2R 1M detector with an individual pixel size of 75 μm and the test was performed in a vacuum (< 1 mbar) environment.

Preparation of liquid crystal oligomers:

14.44 mol% of the diacrylate liquid crystal monomer RM 82, 1.43 mol% of the diacrylate second-generation molecular motor 1, and 14.29 mol% of the chain extender dithiol EDDET were added to a vial, and then the mixture was dissolved using methylene chloride (DCM). When the solution was homogeneous, 69.84 mol% of the triethylamine (TEA) was added dropwise to the solution, and the reaction was carried out at 40 °C overnight. The crude product obtained was diluted with DCM, and the organic layer was washed with 1 M aq. HCl (2 × 100 ml) and saturated brine to remove TEA. After drying with Na_2SO_4, the solvent was removed in vacuum to obtain liquid crystal oligomers. Finally, the oligomer (98 wt%) was mixed well with photoinitiator IRG 819 (2 wt%) in DCM, and the solvent was removed in vacuum and protected from light until use. When DBU was used as a base catalyst instead of TEA, the molar contents of monomer RM 82, motor M1, EDDET and DBU were 37.7 mol%, 3.73 mol%, 37.27 mol% and 21.3 mol%, respectively.

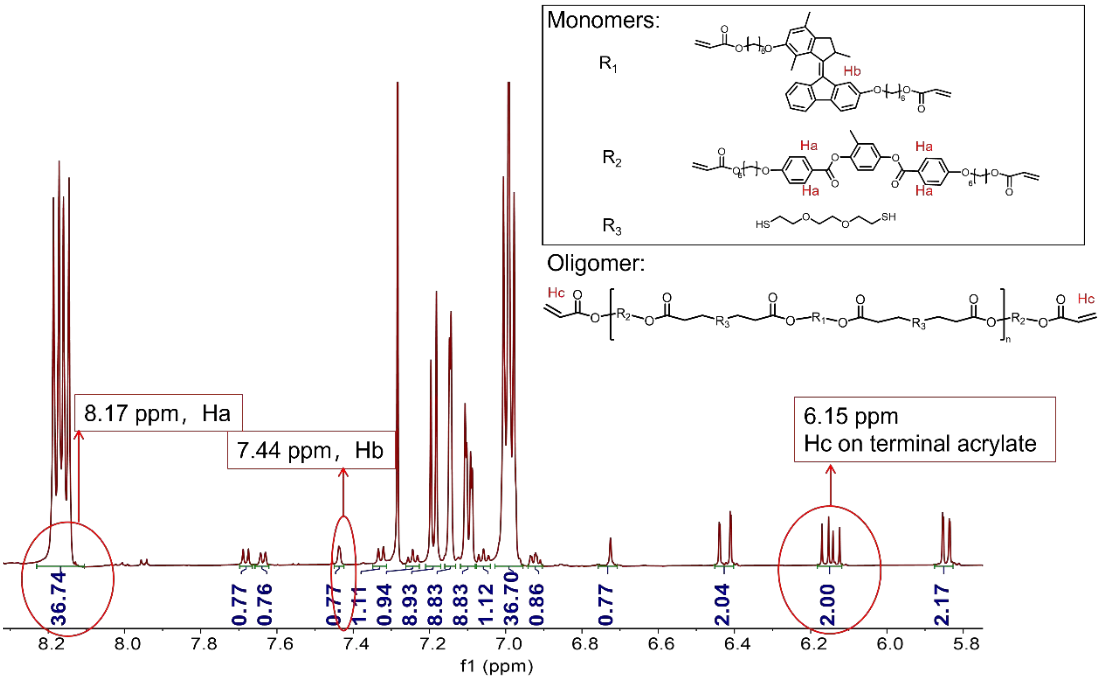

Figure S1 Partial ^{1}H-NMR of an oligomer with an acrylate-thiol ratio of 1:0.9. The absorption at 8.17 ppm is the peak of hydrogen on the benzene ring of the liquid crystal monomer RM 82 (labeled Ha), then the absorption at 7.44 ppm is the hydrogen on the benzene ring in the lower half of the molecular motor (labeled Hb), and the absorption at 6.15 ppm is the peak of the hydrogen in the functional group of the acrylate at the end of the oligomer (labeled Hc).

Calculation of the degree of polymerization of the oligomer:

By integrating the three groups of characteristic absorptions of Ha, Hb, Hc, respectively, and set the integral area of the hydrogen atom on the acrylate (labeled as Hc) to 1, and the absorption areas of the three groups of peaks are noted as Sa, Sb and Sc. Then the degree of polymerization of the oligomer can be calculated by the following formula:

$$DP = \frac{Sa}{2Sc} + \frac{2Sb}{Sc}$$

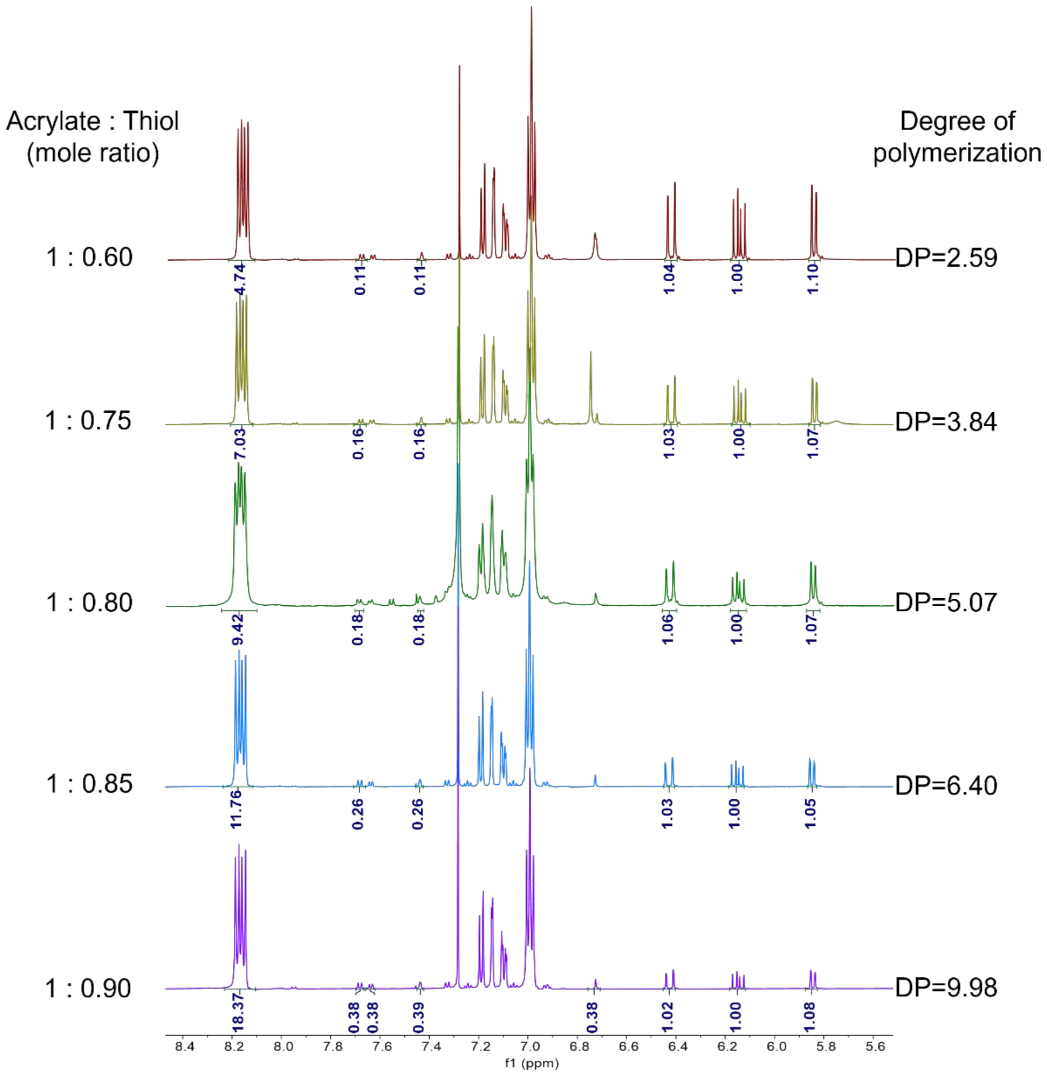

Figure S2 Partial ^{1}H-NMR of acrylate-thiol oligomers with different ratios (1:0.60, 1:0.75, 1:0.80, 1:0.85, 1:0.9) and degree of polymerization of each ratio calculated from the spectra (DP=2.48, 3.68, 4.89, 6.14, 9.60).

Differential scanning calorimetry (DSC):

Study of the phase behavior of liquid crystal oligomers and polymers.

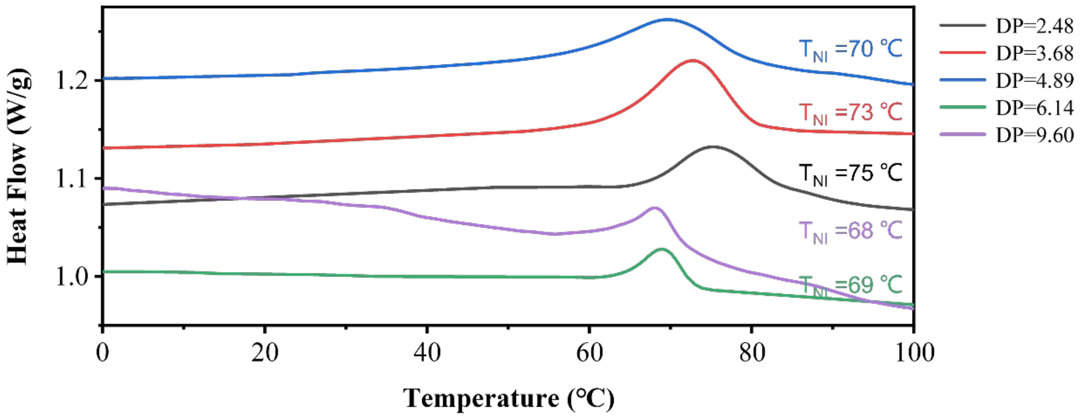

Figure S3 DSC heating up curves of oligomers with different degrees of polymerization and the phase transition temperatures (T_{NI}) corresponding to each degree of polymerization. The heating rate is 5°C/min.

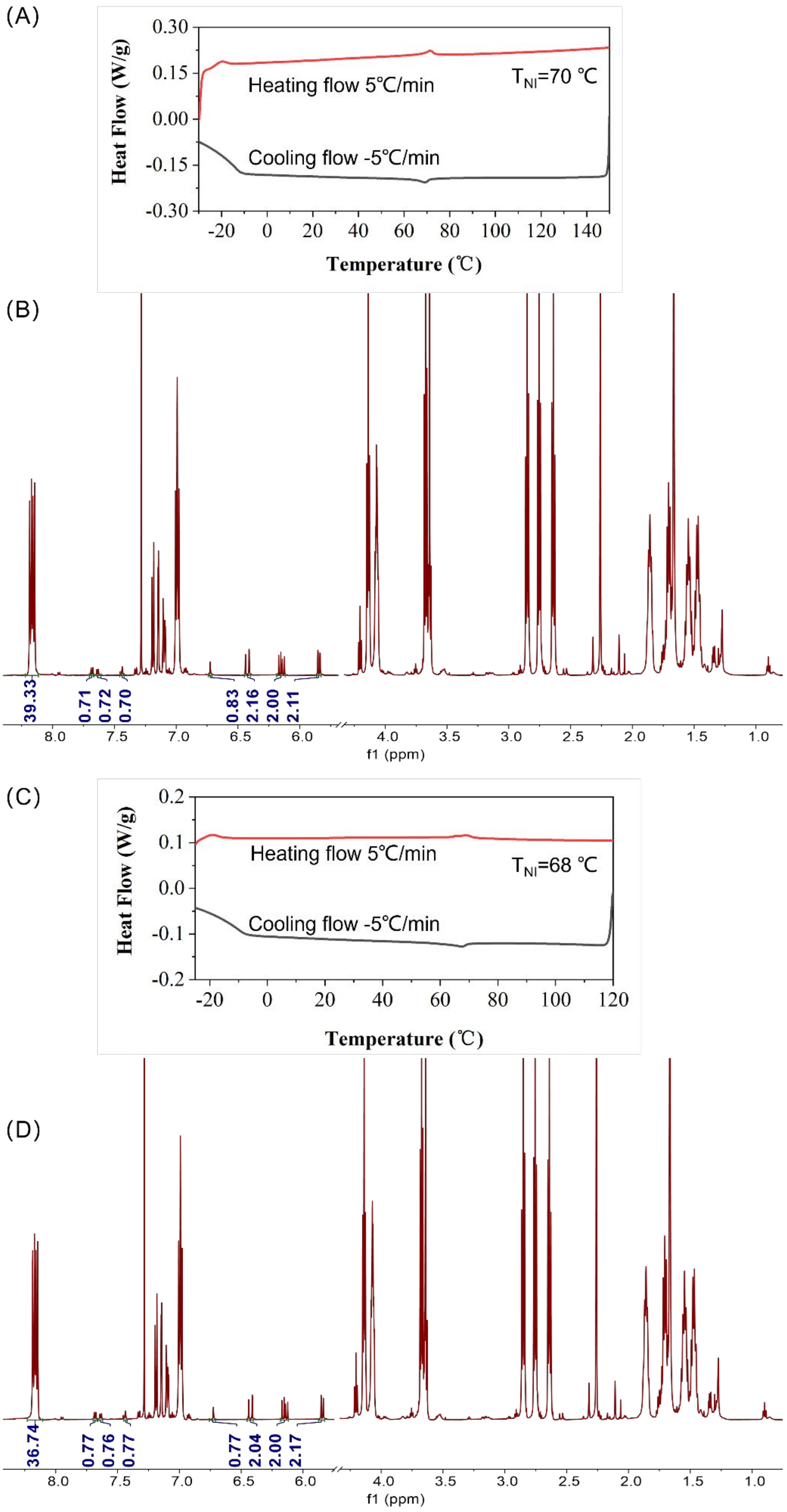

Figure S4 (A) DSC curves of liquid crystal oligomers obtained using DBU as a base catalyst. (B) ^{1}H-NMR of liquid crystal oligomers obtained using DBU as a base catalyst.

(C) DSC curves of liquid crystal oligomers obtained using TEA as a base catalyst. The sample was heated from -30 to 150 ℃ with a heating rate of 5 ℃/min and then cooled down to -30 ℃ with a cooling rate of -5 ℃/min. (D) ^{1}H-NMR of liquid crystal oligomers obtained using TEA as a base catalyst.

GPC (Gel Permeation Chromatography) was used to evaluate the molecular weight of liquid crystal oligomers:

An Agilent PL gel 5 µm M IXED-C (manufactured by GB) column was used with chloroform as the mobile phase at a flow rate of 1 ml/min and a temperature of 35 °C. The reference material was polystyrene (PS) with an average Mw of 350,000 g/mol.

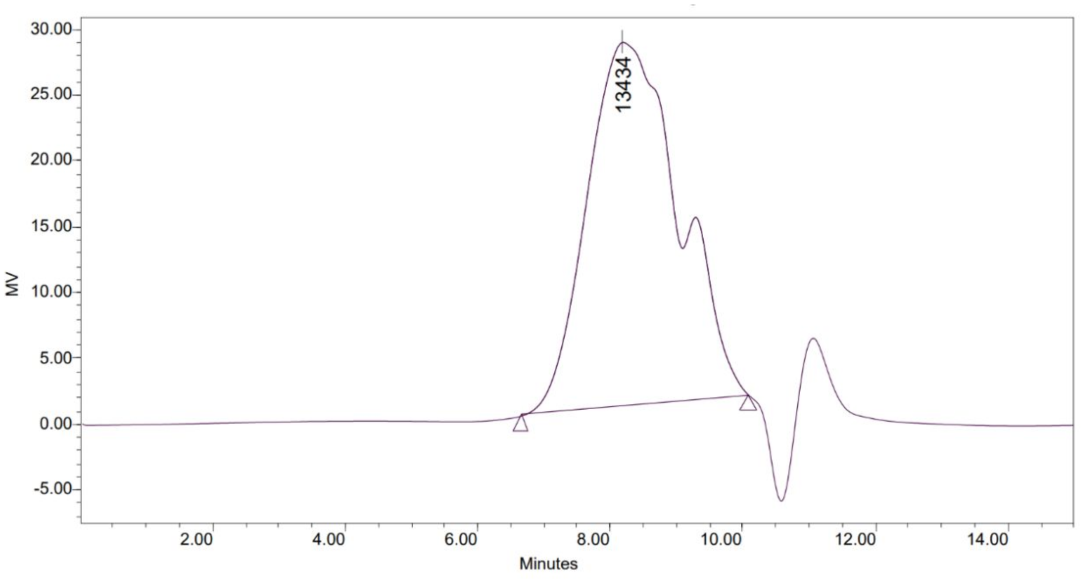

Figure S5 GPC curves with an acrylate-thiol ratio of 1:0.9.

Rheological testing was used to characterize the state of an oligomer to determine if it was suitable for 3D printing:

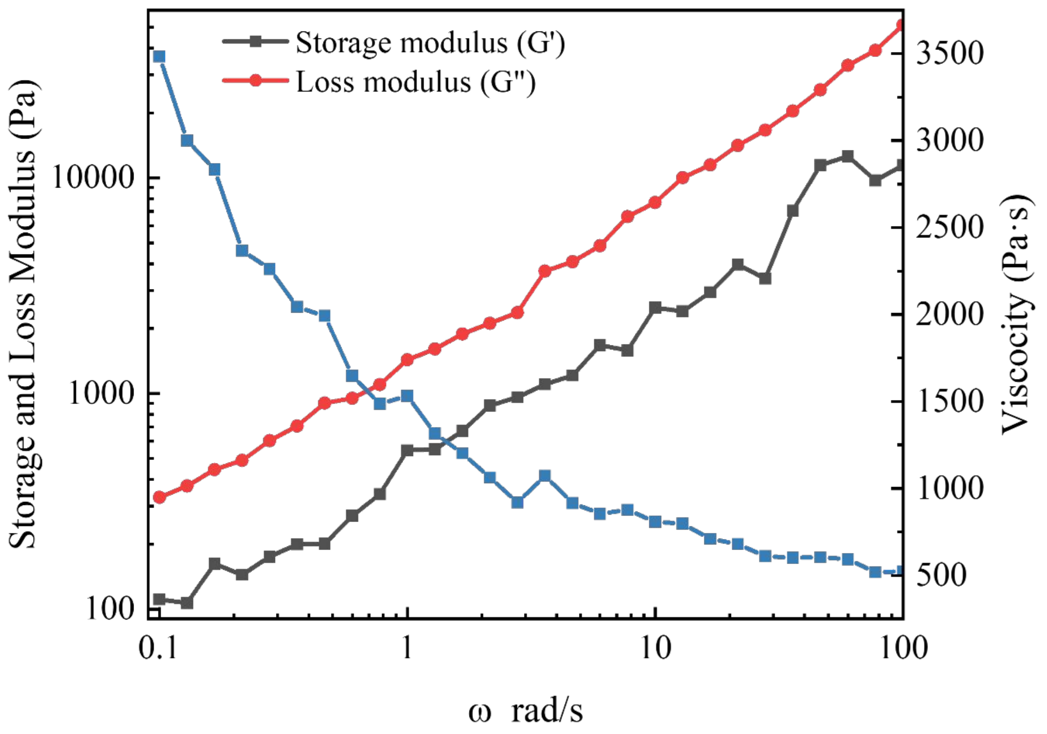

Figure S6 Rheological test. Frequency scanning of the storage modulus (G') and loss modulus (G") at 45 °C. The loss modulus was consistently higher than the storage modulus, indicating that the oligomer was in a fluid state at 45 °C, proving that the conditions were suitable for 3D printing. Black line is storage modulus, red line is loss modulus blue line is viscosity.

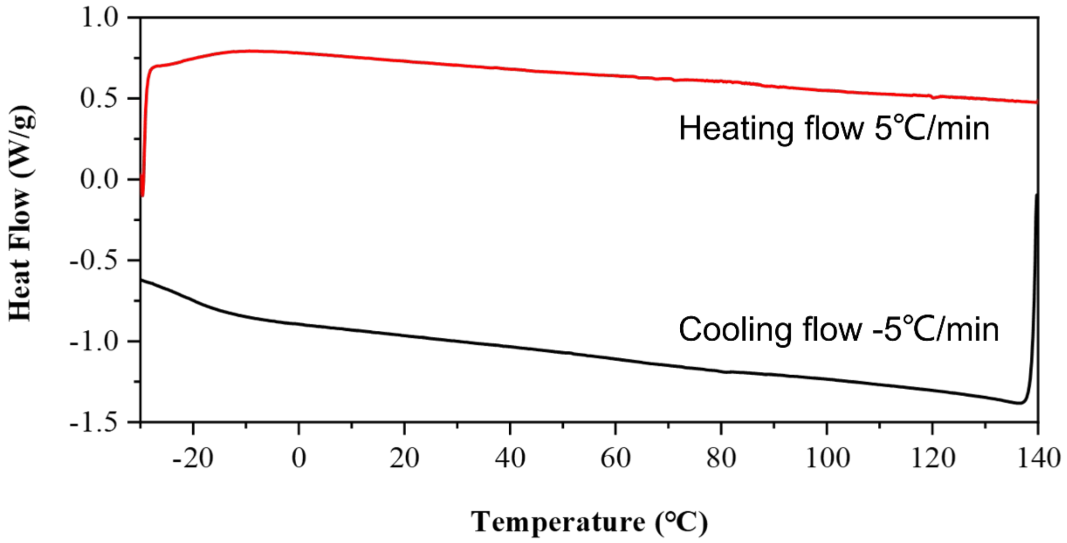

Figure S7 DSC curve of polymeric LCE film. The sample was heated from -30 to 140 ℃ with a heating rate of 5 ℃/min and then cooled down to -30 ℃ with a cooling rate of -

5 °C/min.

FTIR testing was used to characterize the crosslinking state of the liquid crystal mixtures:

From the liquid crystal mixtures to the oligomers, the disappearance of the -SH peak at 2570 nm-1 indicates that there is no -SH present in the end groups of the oligomers and that the initial cross-linking is complete. From the oligomer to the liquid crystal polymer, the disappearance of the C=C peak at 810 nm-1 indicates that the end groups of the acrylates are fully crosslinked, which proves that the printed LCE is fully polymerized.

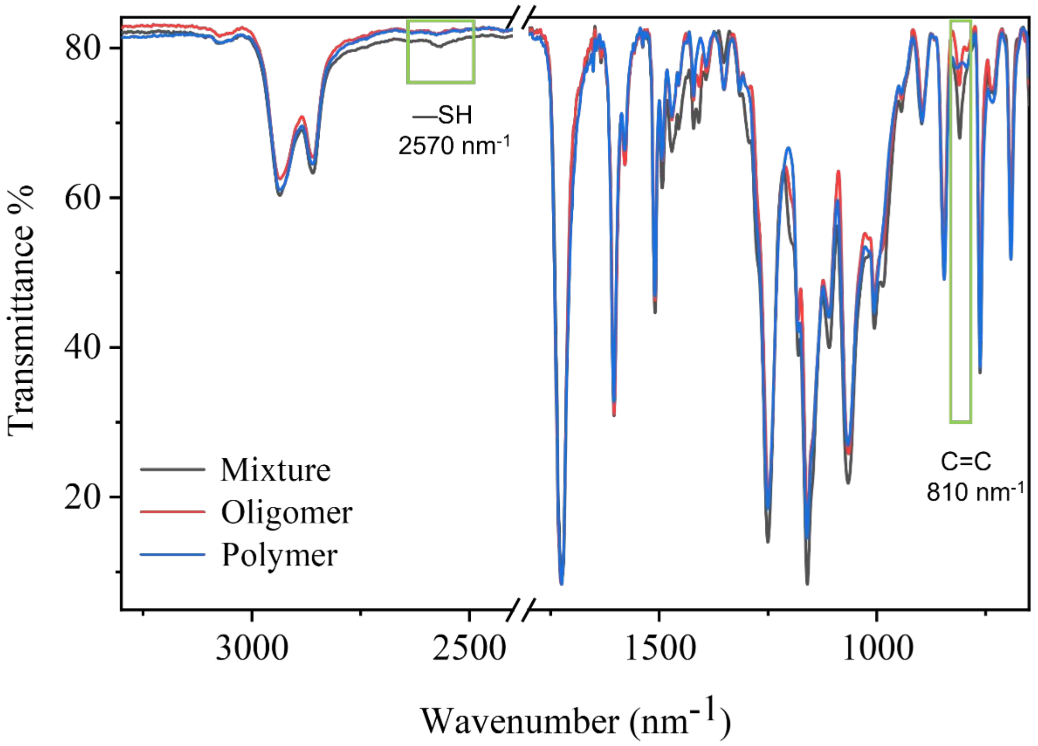

Figure S8 Infrared spectra of liquid crystal mixtures, oligomers, and polymers.

3D printing to prepare liquid crystal elastomer with racemic motors:

The glass substrate was thoroughly cleaned and spin-coated with a polyvinyl alcohol solution (5 wt% PVA aqueous solution). A sacrificial layer was formed by removing all moisture at 100 ℃. Samples were extruded and printed using the EnvisionTec 3D-Bioplotter. The system is equipped with one high-temperature cartridge and two low-temperature cartridges (< 70 °C) and operates through an air pressure of 0-5 bar. To create the samples, the resulting liquid crystal oligomer ink was transferred into a cryogenic syringe cartridge and dispensed through a needle with an inner diameter of

410 μm. The modeling of the three-dimensional structures was constructed by Shapr 3D and sliced using Perfactory software. The barrel temperature was controlled at 45 °C, and the system was held at this temperature for 30 min prior to printing to allow the system to reach stable operating conditions. The substrate temperature was room temperature, the extrusion pressure was controlled at 3 bar, the nozzle diameter was controlled at 410 μm, and the distance between the nozzle and the printed substrate was controlled at 0.6 mm. A UV light source of 455 nm was used at a focal length of 3.0 cm and after each layer at an 100 mW/cm^2 intensity for an initial light curing of the printed structures for 300 s. After printing, photopolymerization was induced using a 200 mW/cm^2 mercury lamp (Exfo Omnicure S2000) with an exposure time of 2 h. The samples were turned over every 30 min to ensure complete cross-linking. The lamp was equipped with a cut-off filter and could only transmit light at a wavelength of 455 nm. To obtain independent samples, the samples were immersed in water at room temperature to dissolve the PVA layer on the glass substrate and subsequently dried overnight at room temperature.

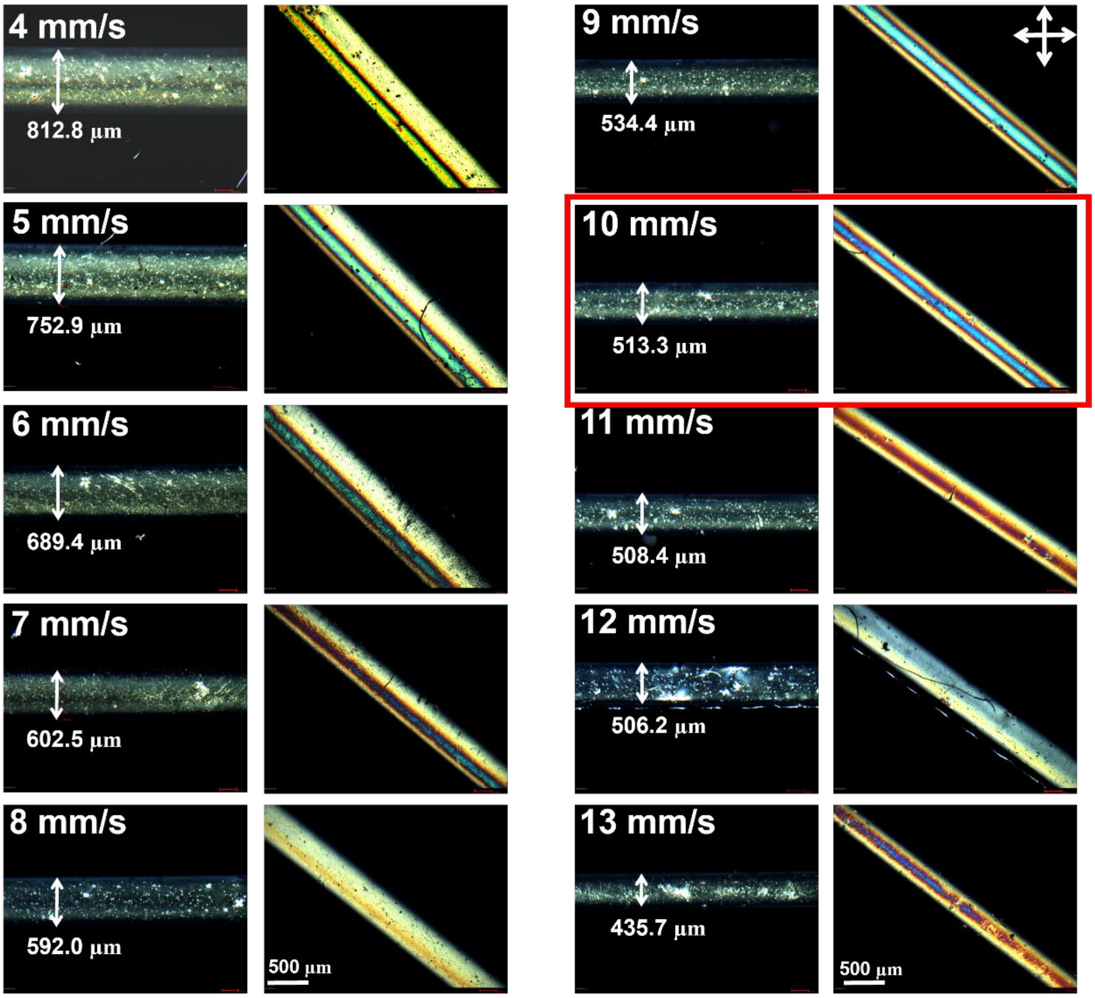

Figure S9 Crossed polarizer micrographs of the lines printed at 45 °C, with a nozzle diameter of 410 μm at different speeds (4-13 mm/s). The white arrows in the upper

right corner indicate the directions of the polarizer and the analyzer for each column. The strips appear bright when the crossed polarisers are oriented at 45°/135°, and dim when they are oriented at 0°/90°. As the print speed increases, the smaller the diameter of the printed strip, the better the orientation. Scale bar represents 500 μm.

Wide-Angle X-Ray Scattering test was used to characterize the internal alignment of liquid crystal elastomers:

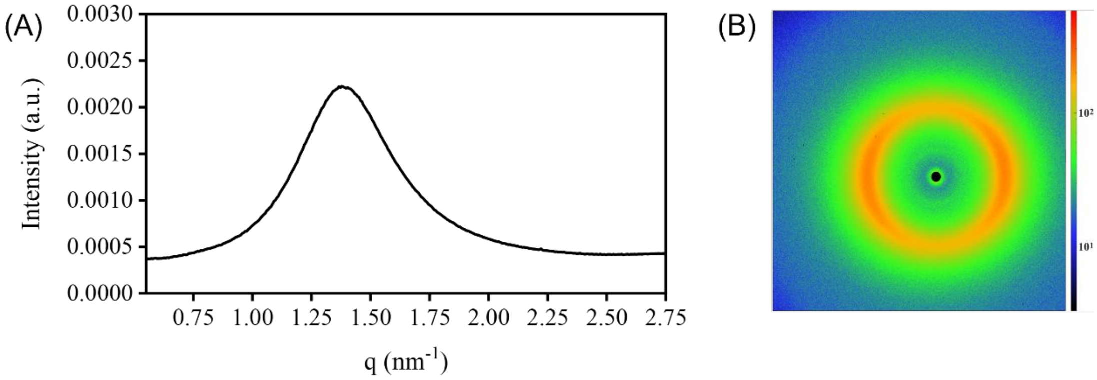

Figure S10 (A) 1-D WAXS of planar aligned LCE film at room temperature. The peak observed at 1.38 nm^{-1} indicates an order. (B) 2-D WAXS pattern of planar aligned LCE film. It exhibits significant intensity differences, characteristic of a typical nematic liquid crystal phase.

Control experiments:

Comparison of the actuation effects of LCE strips in air and under submerged conditions. The ribbon was brought into water and actuation was observed after UV irradiation. It indicates the actuation is driven by rotary motion of molecular motor inside and not by the photothermal effect.

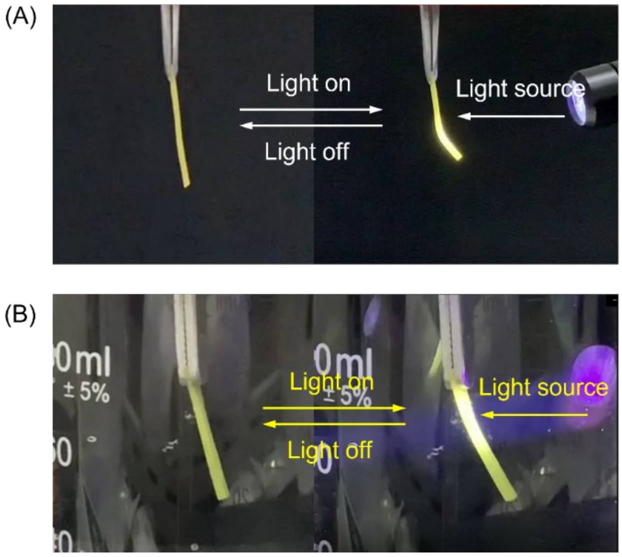

Figure S11 (A) The deformation of LCE ribbon under 365 nm irradiation in air. (B) LCE ribbon based on molecular motors before and after UV irradiation at r.t. under water.

Thermal response deformation of the LCE actuator:

When heat alone was used as a stimulus source to drive the LCE actuator, significant differences in deformation were observed.

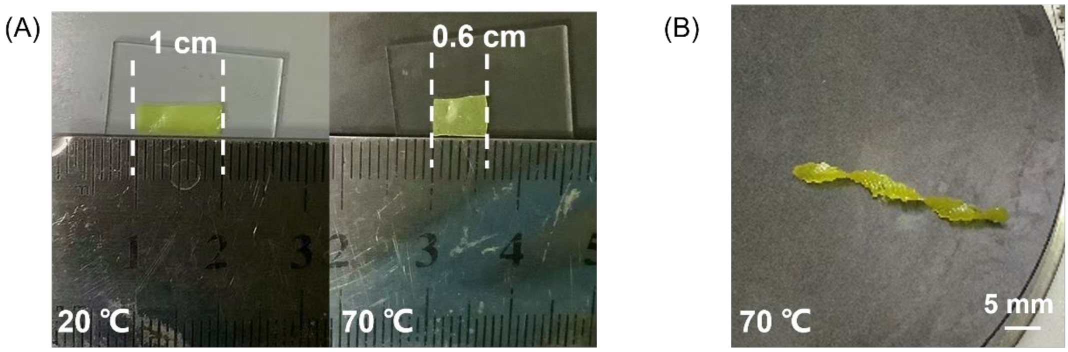

Figure S12 Thermal response effect of LCE actuator. (A) Planar aligned strip was driven by heat to contract in the alignment direction and expand in the vertical direction. (B) 45°/135° strip undergoes twisting deformation driven by heat.

Preparation of liquid crystal elastomer containing chiral motors:

The enantiomeric motor with 5 mol% (containing 1 mol % (R) or (S)) was added to a vial with 50 mol% of liquid crystal monomer RM 82 and 45 mol% of chain extender dithiol EDDET and the mixture was solubilized with DCM. Next connected by slow dropwise addition of the base TEA reacting overnight at 40 °C. The crude product obtained was diluted by DCM, and the organic layer was washed with 1M aq. HCl (2 × 100 ml) and saturated brine was washed to remove TEA, then dried with Na_2SO_4 and

the solvent was removed in vacuum to obtain the CLC oligomer. Finally, the oligomer (98 wt%) was mixed with photoinitiator IRG 819 (2 wt%) in DCM, and the CLC ink was obtained by removing the solvent in vacuum and protecting it from light. For printing, the extrusion pressure was 3 bar, the nozzle diameter was 410 μm, the distance between the nozzle and the print substrate was 0.6 mm, the temperature of the syringe was set to be 90 °C (higher than T_{NI}), and the temperature of the print substrate was set to be 40 °C (lower than T_{NI}) the print speed was 6 mm·s^{-1}. After printing, the samples were placed at 40 °C and irradiated with blue light (455 nm) with a power of 200 mW/cm^2 for 2 h to obtain CLCE films.

Preparation of LCE actuators:

The printing conditions were all 3 bar extrusion pressure, nozzle diameter of 410 μm, distance between nozzle and print substrate of 0.6 mm, syringe temperature of 45 °C, and print substrate temperature of room temperature. After printing, they were exposed to blue light (455 nm) at a power of 200 mW/cm^2 for 300 s to initially fix the molecular arrangement of the liquid crystal. The second layer was then printed and exposed to blue light (455 nm) at 200 mW/cm^2 power for 2 h to obtain the target object.

Design of a bislayer LCE strip:

The size of the bilayer film was set to be 5 mm × 30 mm, the angle of the first layer was set to be 45°, and the internal line spacing of each layer of the sample was set to be 1 mm, which was exposed to blue light (455 nm) at a power of 200 mW/cm^2 for 300 S after printing to initially immobilize the molecular arrangement of the liquid crystals. Then the second layer with an angle of 135° was printed on top of that. Finally, it was exposed to blue light (455 nm) at 200 mW/cm^2 power for 2 h to obtain the LCE bilayer film.

Design of the flower:

The size of each petal of the open and closed flowers was designed to be 5 mm × 15 mm, and the line spacing inside the sample was preset to be 1 mm. one layer of petals with an angle of 0° was set up, and a second layer of petals with an angle of 90° was printed 300 seconds after the initial polymerization.

The size of each petal the spiral flower was designed as 5 mm × 15 mm, the internal line spacing of the sample was preset to 1 mm, a layer of petals was set at an angle of 45°, and the second layer of the structure was printed at an angle of 135° after 300 s of initial polymerization.

Design of the butterfly:

The 3D butterfly was printed in three layers according to the designed pattern and the three layers were printed in the same path. The bionic butterfly can be obtained by splicing with the support material PCL, whose printing parameters are shown in Table

S12.

Support material : Polycaprolactone (PCL)

Molecular Weight	50,000 – 90,000 Da
Ink	Dissolved in dichloromethane
Processing Temperature	25 ℃
Temperature of glass substrate	40 ℃
Needle diameter	340 µm
Pressure	2 bar
Printing speed	15 mm/s

Table S13 Printing parameters for the support material PCL.
